# Therapeutics

**Published:** 1876-12

**Authors:** 


### I. Therapeutics.

1. *On the Methods of Elimination and on the Elective Action of Quinine.* Albertoni and Ciotto. (*Phil. Med. Times*, Sept. 2, 1876.)

Under this title Drs. A. and C. contribute the details of certain experiments made by themselves to the Bull. Gén. de Thérap., No. 8 and 9, 1876. They show—

1. That the presence of quinine in the bile may be demonstrated in from two to five hours after its introduction into the stomach.

2. The elimination of quinine by this path is quite active, since it is found only two hours after ingestion, and after the ingestion of sixty centigrammes (nine grains) only. It is found also that in the dog it produces in twenty-four hours an average secretion of four hundred grammes of bile.

3. Though quinine may not be found in the bile sometimes until two and a half hours after ingestion by the mouth, this is not against the fact that its proper route for elimination is through the hepatic secretion, since, as is known, this is much less rapid than elimination by the urine.

To MM. Albertoni and Ciotto the positive result that the biliary secretion contains quinine, administered by the mouth, appears important. The common mode of entrance of quinine, as of almost all medicines, is by the digestive tube. In the stomach its absorption is favored by the acids present, particularly by the hydrochloric acid, while in the intestine, on the contrary, this is rendered less easy by the alkalinity of the enteric and pancreatic secretions, and still less by the biliary acids which form insoluble compounds with quinine, though these last are soluble in excess of acid. Thus the absorption of quinine in the intestine only takes place sparingly under physiological conditions. Once entered by gastro enteric absorption into the portal circulation, quinine finds a natural anatomico-physiological route for elimination in the biliary secretion. This fact the experiments of Messrs. A. and C. positively demonstrated.

The presence of quinine in the biliary secretion serves to establish the important fact of its electivity of action, since it shows how this alkaloid places itself in intimate contact with the hepatic cells, constituting the functionating element of the liver, of which the bile is the complex product of secretory elaboration. Quinine, therefore, introduced by alimentary passages, appears to stop by preference in the liver and the spleen.

As regards the question whether quinine introduced into the circulation by other routes than by the portal system, is eliminated by the bile or by the urine alone, Messrs. Albertoni and Ciotto find that, hypodermically injected, quinine is eliminated by the urine — an important fact in practice, for it is thus useless to administer the remedy by this method if we expect to affect the liver and spleen. Quinine taken by the mouth is in part eliminated directly by the portal circulation without passing into the general venous system. As regards the length of time during which quinine remains in the organism, it has been found in the urine sixty-eight hours after ingestion. Finally, quinine was always found by MM. A. and C. in the spleen; nearly always in the liver, viscera in which it remains for the longest time. In the heart, quinine is found in larger quantity when introduced hypodermically than when taken by the mouth. In the brain it appears very quickly, but in smaller quantity than in the other viscera mentioned. J. S. K.

2. *Vomiting in Pregnancy cured by Hyoscyamia.* Pitois. (*Phila. Med. Times*, Sept. 2, 1876.)

Dr. P. reports (*Med. Press and Circ.*, May 19, 1876,) two cases of this. After trying, unsuccessfully, all the usual means, it occurred to him to administer a teaspoonful every hour of a mixture containing five milligrammes of hyoscyamia in one hundred and twenty-five grammes of fluid. The next day the vomiting ceased, did not recur, and the patient went on favorably to the natural term of her pregnancy. A second case of the same kind was cured by the same remedy. J. S. K.

3. *Bisulphide of Carbon in the treatment of Cancer of the Stomach.* Whittaker. (*Clinic.*, Aug. 12, 1876.)

Dr. W. reports two cases of undoubted cancer of the stomach much benefitted by the drug. Accepting the view that cancer is a dyscrasia, like tuberculosis, the doctor attributes the benefits to the solvent powers of the drug, and the relief of pain to its anæsthetic properties.

"The bisulphide of carbon, discovered by Lampadius, is a transparent, colorless, very volatile liquid, of great refractive and dispersive power, of a pungent, somewhat aromatic taste, and a peculiar odor, which when pure slightly resembles that of chloroform. Its vapor explodes when mixed with air, so that great care must be taken not to drop it near a light. Carbon bisulphide is miscible in all proportions with absolute alcohol, ether, chloroform, benzol, essential and fatty oils. It dissolves readily and freely among other substances, sulphur, phosphorus, bromine, iodine, iodoform, camphor, gutta percha, resins, wax paraffine, stearine, chloral hydrate, and those alkaloids which are soluble in ether and alcohol. When pure, its odor should not be fetid nor repulsive; it should not cause a dark precipitate in a solution of plumbic acetate, nor change the color of moist litmus paper. Its specific gravity is 1.27 at 15° C."

The doctor administered the drug in two to four drop doses, three times daily, in a teaspoonful of alcohol or almond oil. J. S. K.

4. *New Use of Quinine.* (*The Doctor*, September, 1876.)

The *Clinic* quotes from the *South. Med. Record* the report of a new use of quinine, founded upon its well-known influence on the white corpuscles, and therefore upon suppuration. In the first case six grains of quinine, dissolved in two-thirds of an ounce of water, was injected into the cavity of a suppurating pleura, and the discharge of pus rapidly diminished. An ointment of quinine (10 gr. to ℥j.) was applied to an ulcer on the leg, of two years standing, and associated with initial heart disease. In two or three days suppuration diminished, then healthy granulations appeared, and the ulcer was rapidly healed. The third experiment was on a mammary abscess, treated by the injection of quinine (grs. 10 to ℥j. of water).

J. S. K.

5. *Powder to prevent Pitting after Variola.* Sennabaria. (*The Doctor*, September, 1876.)

Dr. S., of Ragusa, having successfully treated several cases of eczema and acne with a powder composed of four parts of flour of sulphur and one part of red precipitate (*Medical Record*, New York), was induced to try the same in variola. He painted the pustules first with glycerine at the period of suppuration, then dusted the powder over them. A crust forms which eventually falls off, and leaves the skin exempt from cicatrices. J. S. K.

6. *A New Adhesive Plaster.* (*Med. and Surg. Reporter.*)

Mix twenty parts mucilage and one part of glycerine. Spread three or four layers on linen—allow to dry. It may be kept more than a year without deterioration, and is much cleaner and cheaper than diachylon.

J. S. K.

7. *Salicin and Salicylic Acid in Rheumatism.* MADAGAN. (*Brit. Med. Jour. Cin. Lancet and Observer*, September, 1876.)

Dr. M. compares the merits of these two remedies. That each exercises a marvelous influence in cutting short an attack of acute rheumatism, there can be no doubt. I have used salicin or salicylic acid in every case of acute rheumatism which has come under my care since November, 1874 (a year and a half), and invariably with the same result—a rapid cure of the disease. Seeing a patient suffering from acute rheumatism, I have no hesitation in assuring him that within forty-eight hours, possibly within twenty-four, he will be free from pain.

The chief objection to salicylic acid is its tendency to produce irritation of the throat and stomach. I may have been unfortunate in my experience, but in every case in which I have given it this irritation has been complained of. All writers on this subject agree in referring to this irritation as one of its unpleasant effects. Salicylate of soda seems to give rise to the same disgreeable symptom. Salicin, on the other hand, never gives rise to any unpleasant effects. Salicin is a pleasant bitter, and is best given mixed with a little water, flavored with syrup of orange if desired. In adequate doses, say fifteen grains every two hours, it cuts short an attack of rheumatic fever, without producing disagreeable effects. It should be continued in smaller doses during the first fortnight of convalesence. J. S. K.

8. *Quinine Bromohydrate.* GUBLER. (*Jour. de Thèr.; Detroit Review.*)

From a study of this drug G. concludes:

1. The bromohydrate of quinine (bromide of quinine) corresponding to the sulphate of the same base, is more soluble and contains more of the alkaloid than the latter.

2. It possesses the physiological properties of the salts of quinine in general, and probably also the therapeutic virtues of its officinal congener.

3. The action of the bromohydrate of quinine seems, however, to differ from that of the sulphate, not only in the slight degree of cinchonism produced by it, but also by a marked tendency to nervous sedation and hypnotism.

4. The combination of these qualities especially adapts it to the treatment of the congestive and febrile affections which attack the nervous system, neuralgias due to neuritis, irritative neuroses, cerebral hyperæmias etc., in the treatment of which it has afforded excellent results.

5. The bromohydrate of quinine was remarkably efficacious in a case of intractable vomiting. It has proved of great service in a number of affections ordinarily amenable to the action of the sulphate of quinine, visceral or articular congestions—whether of diathetic origin or not—rheumatic or gouty symptomatic fevers, or fevers *a frigore*, etc.

6. The remedy has been given in doses of from six to fifteen grains a day in divided doses of three grains at a time, either in the form of pills or by hypodermic injection. J. S. K.

9. *Chloral Plaster.* (*Lancet.*)

For neuralgia, rheumatic pains, etc., use the ordinary emplastrum roborans,* and powder it with the chloral, apply the plaster to the affected part and leave it from twenty-four to forty-eight hours. When taken off the skin will be found studded with vescicles. These are to be pricked with a pin, followed by a dressing with simple ointment. The pain vanishes long before the vesicles are dried up.

10. *Salicylic Acid for the Profuse Perspiration of the Feet.* C. KUESTER. (*Deutsche Zeitschr. f. pract. Med.;* Centralztg No. 82, 1876.)

Dr. K. thinks the influence of the salicylic acid on the above disorder is equalled only by its wonderful effect in articular rheumatism. He uses the following powder: ℞ Acid. Salicyl. ʒ ij; Talc. Praep. ℥ ss; Amyl. ʒ iiss; Sapon. ʒ j. After the feet have been thoroughly washed and dried the powder is dusted over them and between the toes, and some of it is put in the stockings. In all cases so treated, within the past year, the cure was a permanent one. After two or three applications the bad odor disappeared; the secretion of sweat was considerably diminished in quantity, and the epidermis hitherto continuously moistened and macerated by the perspiration, became dry and could be removed in large shreds, under which was developed a new skin of an entirely normal appearance. If this treatment was discontinued after a few months, the excretion of sweat remained reduced in quantity *without being entirely suppressed.*

In a correspondence with the *Zeitschrift*, Dr. Page speaks very highly of the gratifying success he has had by treating the profuse prespiration of the feet with the following powder, viz.: ℞ Acid. Salicyl. gr. x; Acid. Tannic. gr. xv; Talc. Praepar. and Pulv. Irid. Florent. *aa* ℥ ss. M. The daily application of this powder between and under the toes and in the stockings or socks stopped the maceration of the skin, and fœtid odor attending it, and reduced the excretion of sweat to the normal standard.

11. *Sulphate of Cinchonidia.* BATLEY. (*Atlantic Med. and Surg. Jour.*, Oct., 1876.)

Dr. B., during the last five months, has largely used the above salt as a substitute for quinia sulphate. He finds it has the same antiperiodic and antipyretic properties of quinia sulphate, seldom produces tinnitus aurium, but is more inclined to nauseate a delicate stomach. For country physcians who furnish their own medicines it is decidedly cheaper than quinine.

A good way of administration to children is to make an acidulated solution of the salt, then precipitate the cinchonidia with ammonia. And administer the washed and dried precipitate with equal parts of liquorice powder. Owing to its insolubility it is almost tasteless. J. S. K.

12. *Bromohydric Acid.* FOTHERGILL. (*British Med. Journal.*)

Dr. F. obtains the acid by dissolving ten ounces, six drachms, twenty-

* Emp. oxidi ferri rubri. Strengthening Plaster.

eight grains of potassium bromide in four pints of water, and adding thirteen ounces, one drachm, thirty-seven grains of tartaric acid. The bitartrate of potash is precipitated and the hydrobromic acid remains in a clear, bright, almost colorless fluid, possessing an acid taste and the ordinary acid properties as well as the peculiar properties of potassium bromide, as compared with any other salt of potash. It prevents the headache following a dose of quinine, of which salt it is an excellent solvent. It is peculiarly useful in hysterical conditions, associated with ovarian excitement, the vomiting of pregnancy, and all reflex excitements. With quinine it fulfills the same indications as bromide of potassium in such diseases as whooping cough, and excitement from nervous exhaustion. With chloroform and syrup of squills, it is a palatable and efficient cough mixture. Full dose, prepared as above, one drachm.—*Med. and Surg. Reporter*, Oct. 14, 1876. J. S. K.

13. *Oxygen Gas.* HEARD. (*Atlanta Med. and Surg. Journal*, Oct., 1876.)

Dr. H. advocates inhalations of the gas in incipient phthisis, and reports five cases in endorsement. From a review of them it appears that a daily inhalation of four gallons of the the gas removed muco-purulent expectoration and night sweats, improved digestion and increased weight. A brief continuance of treatment cured the cases reported. J. S. K.

14. *Atropia as an Antidote to Hydrocyanic Acid.* JACKSON. (*Druggists' Circular*, Jan., 1876.)

In experimenting on dogs, Dr. J. says: Sulphate of atropia, in doses of one-fourth of a grain to one grain, injected under the skin, gave prompt relief in every case, even when large doses of the acid had been given. When the two poisons are administered at the same time none of the effects of prussic acid are developed; but if as much as a grain of sulphate of atropia be injected, all the symptoms of atropia poisoning are observed. In some instances the antidote was withheld until the animal would fall down, and the respirations would be as few as six per minute, the dog being unconscious, then one-fourth grain of the antidote would relieve him immediately. J. S. K.

